# Solubility and Thermodynamics of Lithium Carbonate in Its Precipitation Mother Liquors

**DOI:** 10.3390/molecules30173617

**Published:** 2025-09-04

**Authors:** Haiwen Ge, Huaiyou Wang, Min Wang

**Affiliations:** 1 Key Laboratory of Green and High-End Utilization of Salt Lake Resources, Qinghai Institute of Salt Lakes, Chinese Academy of Sciences, Xining 810008, China; 2 Qinghai Provincial Key Laboratory of Resources and Chemistry of Salt Lakes, Xining 810008, China

**Keywords:** lithium carbonate, lithium precipitation mother liquor, solubility, E-DH equation, thermodynamic calculation

## Abstract

This study systematically investigated the dissolution equilibrium of lithium carbonate (Li_2_CO_3_) in mixed Na_2_CO_3_-NaCl aqueous solutions through isothermal dissolution experiments spanning 283.15–353.15 K. Precise solubility determinations were conducted using a gravimetric analysis under controlled thermodynamic conditions. The obtained solubility data were successfully correlated with the Extended Debye–Hückel (E-DH) model, yielding residual standard deviations below 0.09, which validates the model’s applicability in this ternary system. Both experimental observations and theoretical predictions confirmed that increasing the salt molality enhances the synergistic suppression of the Li_2_CO_3_ solubility through combined common-ion and salt effects. The thermodynamic analysis revealed the dissolution process to be exothermic (Δ*H_d_* < 0), and entropy change dominates (*ξ_S_* ≈ 78%), with negative entropy changes (Δ*S_d_* < 0) indicating predominant hydration ordering effects. These mechanistic insights establish critical thermodynamic benchmarks for optimizing lithium carbonate precipitation processes in brine lithium extraction operations.

## 1. Introduction

Lithium, a naturally occurring trace element, is widely distributed in geological formations, including igneous rocks, soils, brine deposits, seawater, and biological systems, across plant and animal organisms. Based on incomplete statistical data, current global lithium reserves are estimated at approximately 13 million metric tons (in lithium metal equivalent). Brine lithium resources are primarily concentrated in Bolivia, Chile, China, and the United States [1]. Li_2_CO_3_, a commercially vital compound in lithium salt production, functions both as a flux agent and performance-enhancing additive in glass and ceramic manufacturing. This inorganic salt also serves as a fundamental precursor for synthesizing specialized lithium compounds, including lithium tantalate (LiTaO_3_) and lithium niobate (LiNbO_3_) monocrystals—materials essential for manufacturing surface acoustic wave elements with optimized elastic properties [2,3]. Recent technological developments have revealed significant potential for high-purity lithium salts in emerging applications, particularly within new energy systems, advanced materials, and high-tech industries. These compounds have emerged as critical components in electric vehicle technologies, demonstrating a growing importance in sustainable transportation solutions.

Contemporary lithium extraction primarily focuses on pegmatite-type lithium minerals and lithium-enriched brine deposits, containing lithium-bearing minerals such as spodumene, petalite, lepidolite, and amblygonite. Prior to the 1990s, lithium production predominantly relied on solid mineral processing, a method constrained by high energy consumption, challenging tailings/wastewater management, and significant environmental impacts. Since 1996, salt lake brine extraction has emerged as the dominant global lithium production method, accounting for 80% of the worldwide Li_2_CO_3_ output. Major lithium-bearing salt lakes with proven reserves exceeding one million metric tons include Salar de Uyuni, Salar de Hombre Muerto, Salar de Atacama, and the Zabuye Salt Lake [4,5]. China possesses the world’s second-largest lithium resource reserves after Bolivia, with brine deposits constituting approximately 79% of its total lithium reserves. The Qinghai–Tibet Plateau hosts China’s primary salt lake lithium resources, which demonstrate comparable proven reserves to global counterparts and substantial economic potential, forming a critical foundation for national lithium industry development [6]. Notably, the Qaidam Basin in Qinghai contains brine lithium reserves of 15.201 million metric tons (as LiCl equivalent) [7].

Conventional brine lithium extraction techniques include precipitation, calcination-leaching, selective membrane separation, adsorption, and solvent extraction methods [7,8,9,10,11,12,13]. The industrial process involving LiCl production from brine followed by reaction crystallization with Na_2_CO_3_ to precipitate Li_2_CO_3_ faces persistent challenges: a low lithium yield, product purity limitations, a poor crystal morphology, and severe particle agglomeration. The Li_2_CO_3_ precipitation process represents a critical yet underdeveloped stage in the lithium production chain, combining purification/enrichment with precipitation. While purification techniques receive substantial research attention, precipitation technology remains relatively primitive.

Song et al. [14] systematically investigated Li_2_CO_3_ solubility and supersolubility in aqueous systems through a thermodynamic analysis. Wang et al. [15] extended this work to LiCl-NaCl-KCl-Na_2_SO_4_ mixed solutions, examining salt effects on Li_2_CO_3_’s metastable zone. Cheng et al. [16] determined the solubility of lithium carbonate (Li_2_CO_3_) in Na-K-Cl systems and employed the Pitzer model to fit its solubility in these solutions. Gisele Azimi et al. [17] carried out an in-depth study on the crystallization process of lithium carbonate; when mixing lithium sulfate and a sodium carbonate solution, the sodium sulfate was removed by freezing, the recovery of lithium was 90%, and the purity of the lithium carbonate was 99.0%.

Although significant progress has been made in lithium carbonate preparation using sodium carbonate precipitation, the resulting mother liquor system retains a mixed solution of NaCl and Na_2_CO_3_. Notably, the lithium loss during the carbonation precipitation process reaches up to 15%, highlighting the technical bottleneck that underscores the necessity for systematic investigation into the solubility mechanisms within the lithium carbonate precipitation system. However, fundamental studies on the solubility of Li_2_CO_3_ in Na_2_CO_3_-NaCl aqueous solutions remain critically lacking. This deficiency in phase equilibrium data directly impedes the optimization of lithium recovery efficiency in precipitation processes, despite its pivotal role in advancing industrial-scale lithium extraction. This study systematically examines the solubility of Li_2_CO_3_ in mixed Na_2_CO_3_-NaCl aqueous solutions across a temperature range of 278.15–358.15 K. Experimental solubility data were correlated using the Extended Debye–Hückel equation with subsequent thermodynamic parameter derivation through established thermodynamic modeling. The combined experimental and theoretical investigation aims to elucidate the dissolution behavior of Li_2_CO_3_ in mixed Na_2_CO_3_-NaCl aqueous solutions, while establishing fundamental data to optimize Li_2_CO_3_ precipitation processes.

## 2. Experimental Section

### 2.1. Instruments and Reagents

The main chemical reagents and instruments used in the experiments are listed in Table 1 and Table 2, respectively. All experimental solutions were prepared through direct dissolution of analytical-grade reagents with strict quality control. Secondary deionized water (DDW) with conductivity < 1 × 10^−4^ S·m^−1^ served as the solvent system. Prior to application, the DDW was decarbonized by sequential heating and boiling processes, attaining a stable pH value of 6.60.

### 2.2. Experimental Method

This study employed the isothermal dissolution method [18] to determine lithium carbonate (Li_2_CO_3_) solubility. Sodium carbonate (Na_2_CO_3_) and sodium chloride (NaCl) mixed solutions of varying molalities were separately prepared. Each 150 mL aliquot was transferred to a polyethylene conical flask containing 5 g Li_2_CO_3_ excess solid. The sealed flasks were immersed in a thermostated magnetic stirring bath, maintaining constant temperature (±0.05 °C) with continuous agitation throughout the experiment. Following five-day equilibration with daily sampling verification, supernatants were syringe-filtered through 0.22 μm microporous membranes (25 mm diameter) to eliminate residual particulates. Filtered aliquots were quantitatively transferred to 10 mL polyethylene vials and diluted to 250 mL with deionized water for subsequent analysis. Equilibrium was confirmed when consecutive daily measurements showed <0.5% variation in ionic molalities, at which point the final stabilized values were recorded as solubility data. Solid phases were immediately collected via vacuum filtration to minimize atmospheric exposure. Phase composition of equilibrium solids was verified by X-ray diffraction (XRD) analysis. Powder XRD patterns were collected on a diffractometer using Cu Kα radiation (Kα1 = 1.540598 Å and Kα2 = 1.544426, Ratio K-α2/K-α1 = 0.5) over a 2θ range of 5–80°, with a step size of 0.02° and a counting time of 38 s per step.

### 2.3. Analytical Methods

Lithium ion (Li^+^) molalities were analyzed using inductively coupled plasma optical emission spectrometry (ICP-OES). Carbonate (CO_3_^2−^) molalities were analyzed via standardized hydrochloric acid titration. Chloride (Cl^−^) molalities were analyzed through mercurimetric titration with Hg(NO_3_)_2_ standard solution, employing diphenylcarbazide–bromophenol blue mixed indicator system [19].

## 3. Results and Discussion

### 3.1. Solubility of Li_2_CO_3_ in Mixed Solution

To validate the experimental accuracy of Li_2_CO_3_ solubility determinations, the molal solubility in deionized water was experimentally measured (Table 3). The comparative analysis revealed relative deviations (RDs) below 0.0243 versus the literature values, demonstrating an excellent agreement between experimental and published solubility data.

**Figure 1:**
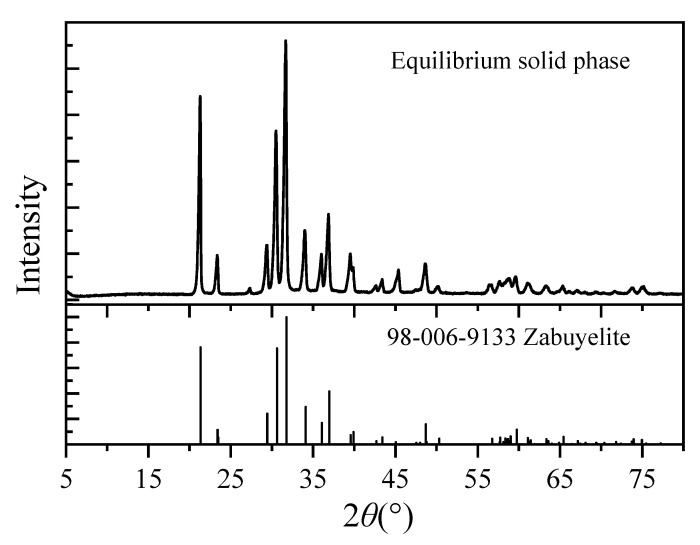
X-ray diffraction pattern of the equilibrium solid phase (Zabuyelite is a natural lithium carbonate mineral).

The solubility of Li_2_CO_3_ in mixed Na_2_CO_3_-NaCl aqueous solutions was experimentally determined across the temperature range of 283.15–353.15 K, with the comprehensive dataset tabulated in Table 4. Figure 1 displays the X-ray diffraction (XRD) patterns characterizing the equilibrated solid phases. The XRD analysis confirmed the absence of Na_2_CO_3_ and NaCl crystalline phases in the equilibrium solids. This observation establishes that the measured molality of Li_2_CO_3_ in the liquid phase directly represents its thermodynamic solubility under the experimental conditions.

**Figure 2:**
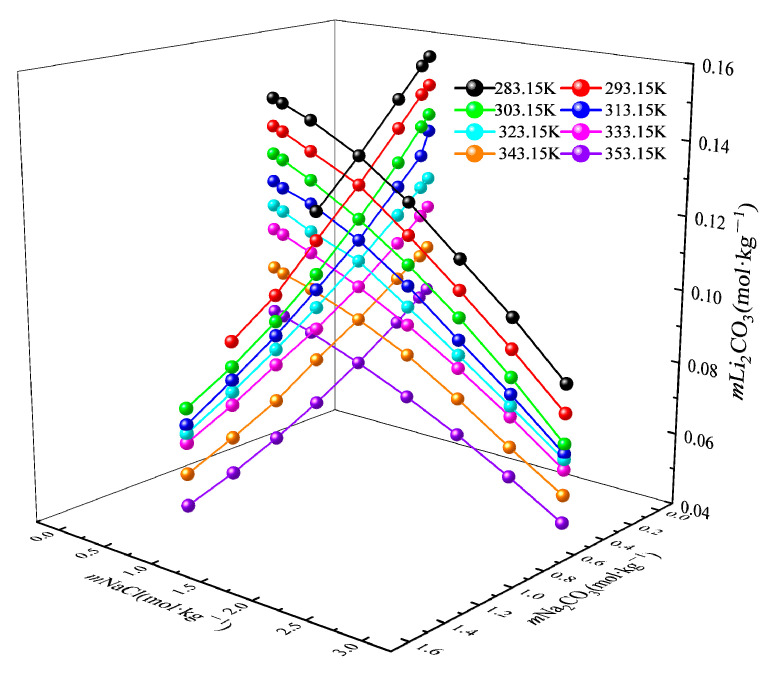
Solubility of Li_2_CO_3_ in mixed solutions of Na_2_CO_3_ and NaCl at different temperatures.

To systematically investigate the influence of the main components in the lithium precipitation mother liquor, Na_2_CO_3_ and NaCl, on the solubility of Li_2_CO_3_, we fixed Na_2_CO_3_ at 0.5 mol·kg^−1^ and NaCl at 1.0 mol·kg^−1^ based on the ionic concentrations in the mother liquor, respectively, while varying the concentration of the other salt. We plotted the Li_2_CO_3_ solubility values as a function of the temperature and the molality of the two salts, as shown in Figure 2. The solubility of Li_2_CO_3_ in the mixed electrolyte system exhibits a clear negative correlation with the temperature, which is consistent with the inverse relationship observed in pure water systems. As the molality of either constituent salt increases, the solubility gradually decreases. This dissolution behavior results from the combined effects of (i) the common-ion effect primarily attributed to the carbonate ions from Na_2_CO_3_ and (ii) the non-common-ion salt effect arising from collective ionic interactions in the mixed electrolyte system.

**Figure 3:**
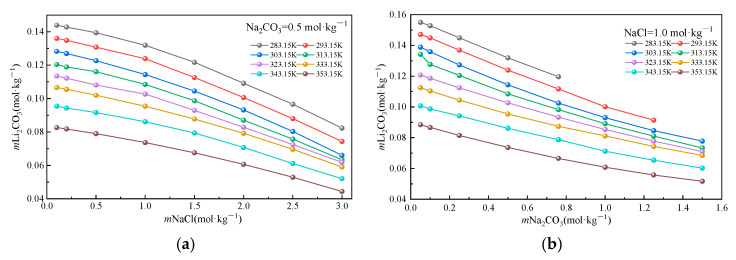
Li_2_CO_3_ solubility in a mixed solution. (**a**): a mixed solution of Na_2_CO_3_ = 0.5 mol·kg^−1^; (**b**) a mixed solution of NaCl = 1.0 mol·kg^−1^.

To isolate the individual effects of Na_2_CO_3_ and NaCl on the Li_2_CO_3_ solubility, Figure 3 illustrates the solubility variations under controlled conditions with incremental salt additions. Experimental data demonstrate systematic decreases in the Li_2_CO_3_ solubility with increasing concentrations of both salts, albeit through distinct mechanisms. As shown in Figure 3a, at a fixed Na_2_CO_3_ concentration (0.5 mol·kg^−1^), the Li_2_CO_3_ solubility decreases monotonically with the rising NaCl concentration. This behavior originates from a non-specific salting-out effect induced by Na^+^ and Cl^−^: Na^+^ compresses the ionic atmosphere surrounding CO_3_^2−^ through electrostatic screening, significantly reducing the activity coefficient of CO_3_^2−^ (γCO_3_^2−^). Simultaneously, Cl^−^ enhances the solution polarity, promoting Li^+^ hydration and decreasing the free Li^+^ concentration. The temperature elevation intensifies this suppression: the reduced dielectric constant (*ε*) of water strengthens the electrostatic attraction between Na^+^ and CO_3_^2−^, further inhibiting the dissolution equilibrium. Figure 3b reveals that at a fixed NaCl concentration (1.0 mol·kg^−1^), the increasing Na_2_CO_3_ concentration also reduces the solubility but with a progressively diminishing rate. This non-linear response stems from competing ion interaction mechanisms: (i) the common-ion effect (CO_3_^2−^) directly suppresses Li_2_CO_3_ dissociation, while high Na^+^ concentrations compete with Li^+^ for CO_3_^2−^ binding, forming NaCO_3_^−^ ion pairs and reducing free carbonate, and (ii) at elevated Na_2_CO_3_ concentrations, a high CO_3_^2−^ density promotes Li^+^-CO_3_^2−^ ion pair formation (LiCO_3_^−^), generating a salting-in effect. Concurrently, the dielectric constant reduction due to the increased ionic strength enhances the Li^+^-CO_3_^2−^ electrostatic attraction, partially offsetting the common-ion suppression. It is particularly noteworthy that the temperature exerts a dominant influence: Li_2_CO_3_ maintains an inverse solubility–temperature dependence across all salt concentrations, which is consistent with its behavior in pure water. Elevated temperatures primarily facilitate ion association by weakening hydration shells while simultaneously reducing *ε*^r^ to intensify interionic electrostatic forces (inhibiting dissolution). These mechanistic insights reveal limitations in industrial precipitation processes: the competitive shielding of CO_3_^2−^ by Na^+^ and the saturation of ion association effects prevent the linear enhancement of the lithium recovery efficiency with excessive Na_2_CO_3_ additions.

### 3.2. Solubility Model

The dissolution equilibrium of Li_2_CO_3_ in mixed Na_2_CO_3_-NaCl aqueous solutions under isothermal conditions is governed by two competing mechanisms: the common-ion (homoionic) effect and ionic strength-mediated salt effects. The common-ion effect modifies the dissolution equilibrium through solubility product (*K_sp_*) constraints, while the salt effect operates via the ionic strength-dependent modulation of activity coefficients for Li^+^ and CO_3_^2−^ ions in the solution. Following the classical solubility product theory, the dissolution equilibrium for Li_2_CO_3_ in this ternary system can be formally expressed as

(1) $$K_{sp}(T)=m_{{\mathrm{Li}}^{+}}^{2}m_{{\mathrm{CO}}_{3}^{2-}}$$

Using experimental solubility data of Li_2_CO_3_ in pure water, Cheng et al. [16] derived new parameters for the Li^+^-CO_3_^2−^ ion pair within the Pitzer activity coefficient model, enabling the successful prediction of Li_2_CO_3_ solubility in NaCl, KCl, LiCl, and NaCl-KCl mixed solutions. While such ion-specific models provide accurate predictions, they require extensive parametrization for complex systems. To develop a more streamlined approach for quantifying concentration-dependent solubility variations across mixed electrolytes, we established reference solubility products (*K_sp_*(*T*)) for Li_2_CO_3_ in defined baseline solutions (0.5 mol·kg^−1^ Na_2_CO_3_ + 1.0 mol·kg^−1^ NaCl). These reference values were subsequently incorporated into an Extended Debye–Hückel (E-DH) framework to construct a predictive solubility model for multi-component systems [20].

(2) $$\mathrm{lg}(\beta )=\mathrm{lg}(\frac{K_{sp}(T)}{K_{sp}^{0}(T)})=-\frac{8A\sqrt{I}}{\left(1+Ba\sqrt{I}\right)}-CI-DI^{2}$$

(3) $$I=\frac{1}{2}\sum_{i}m_{i}Z_{i}^{2}+\frac{1}{2}\sum_{j}m_{j}Z_{j}^{2}$$

(4) $$A=\frac{1}{2.303}{\left(\frac{2\pi N_{A}}{1000}\right)}^{0.5}{\left(\frac{e^{2}}{DkT}\right)}^{1.5}$$

where *I* denotes the ionic strength of the solution at the dissolution equilibrium, mol·kg^−1^; *A* denotes the Debye–Hückel limiting slope, which is calculated by listing *A* in Table 5 at different temperatures; *Bα* is used as a model parameter for the theoretical term (long-range force-acting term); and *C* and *D* denote the model parameter for the short-range force correction term.

Based on the solubility model equation (Equation (2)), multivariate regression analyses were performed to determine the solubility product coefficients (*β*) at various temperatures. This computational approach enabled the derivation of thermodynamic parameters for Li_2_CO_3_ in a mixed electrolyte system containing 0.5 mol·kg^−1^ Na_2_CO_3_ and 1.0 mol·kg^−1^ NaCl across the studied temperature range. The optimized parameters are summarized in Table 5. To evaluate the model’s accuracy, both the relative deviation (*ε*, Equation (5)) and standard deviation (*δ*, Equation (6)) were employed as statistical metrics, with the corresponding computational results presented in Table 5.

(5) $$\varepsilon =(m_{{\mathrm{Li}}_{2}{\mathrm{CO}}_{3}}^{\mathrm{cal}}-m_{{\mathrm{Li}}_{2}{\mathrm{CO}}_{3}}^{\exp})/m_{{\mathrm{Li}}_{2}{\mathrm{CO}}_{3}}^{\exp}$$

(6) $$\delta =\sqrt{\frac{1}{n-1}\sum_{i}{\left(m_{{\mathrm{Li}}_{2}{\mathrm{CO}}_{3}}^{\exp}-m_{{\mathrm{Li}}_{2}{\mathrm{CO}}_{3}}^{\mathrm{cal}}\right)}^{2}}$$

**Figure 4:**
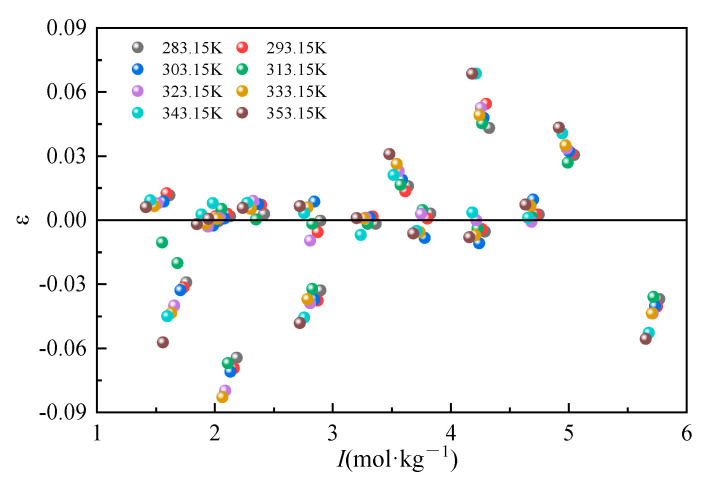
Relative deviation of calculated and experimental values of Li_2_CO_3_ solubility in the mixed system.

The Li_2_CO_3_ molalities in the mixed solution were calculated according to the model parameters, respectively, and the deviation ε relative to the total ionic strength in the mixed solution is plotted in Figure 4. As can be seen in Figure 4, the relative deviations of all the experimental values of the solubility of Li_2_CO_3_ from the model-calculated values are within ±0.1, which suggests that the solubility model equations can better represent the solubility properties of Li_2_CO_3_ in the Na_2_CO_3_-NaCl mixed solution.

### 3.3. Thermodynamics of Dissolution

The solubility of Li_2_CO_3_ in mixed Na_2_CO_3_-NaCl aqueous solutions is expressed in terms of the molar fraction *x*, as in Equation (7):

(7) $$x=\frac{m_{{\mathrm{Li}}_{2}{\mathrm{CO}}_{3}}/M_{{\mathrm{Li}}_{2}{\mathrm{CO}}_{3}}}{\sum_{i}m_{i}/M_{i}}$$

where *m_i_* is the mass of Li_2_CO_3_, Na_2_CO_3_, NaCl, and H_2_O, in grams, respectively; and *M_i_* is the molar mass of Li_2_CO_3_, Na_2_CO_3_, NaCl, and H_2_O, expressed in g·mol^−1^, respectively.

Using enthalpies of formation for solid Li_2_CO_3_ and aqueous Li^+^ and CO_3_^2−^ ions from the literature, Lee et al. [21] calculated the enthalpy of dissolution (Δ*H_d_*) for Li_2_CO_3_ to be −4.3 kJ·mol^−1^ based on a 1 mol/kg standard state via a thermodynamic cycle approach. While such indirect calculations based on formation enthalpies provide valuable insights, the apparent enthalpy of the dissolution can also be directly determined from experimental solubility data using the temperature dependence expressed by the Van’t Hoff equation [22]. This fundamental thermodynamic relationship, derived from activity coefficient relationships under ideal solution approximations, establishes a linear correlation between the natural logarithm of the solute mole fraction (ln *x*) and the reciprocal absolute temperature (1/*T*), as expressed by Equation (8):

(8) $$\ln x=-\frac{\Delta H_{d}}{RT}+\frac{\Delta S_{d}}{R}$$

(9) $$\Delta G_{d}=\Delta H_{d}-\Delta S_{d}\bar{T}$$

where *R* = 8.314 J·mol^−1^·K^−1^ (universal gas constant); Δ*H_d_*= dissolution enthalpy (J·mol^−1^); Δ*S_d_* = dissolution entropy (J·mol^−1^·K^−1^); and *T*= system temperature (K).

The Gibbs energy change (Δ*rG*) calculated here represents the apparent standard Gibbs energy of the dissolution for lithium carbonate (Li_2_CO_3_) in mixed electrolytes. This macroscopic thermodynamic property is derived directly from solubility measurements using the relation Δ*_r_G* = –*RT*ln(*K_sp_*). It should be noted that this approach provides the net thermodynamic driving force of the dissolution equilibrium. The values reported are apparent properties, reflecting the overall thermodynamics of the system under the given experimental conditions, without incorporating molecular-scale solvation studies. Although microscopic analyses—such as solvation energetics (e.g., Δ*G_solv_*)—could offer further mechanistic interpretation, they are not prerequisites for establishing phase equilibrium thermodynamics.

**Figure 5:**
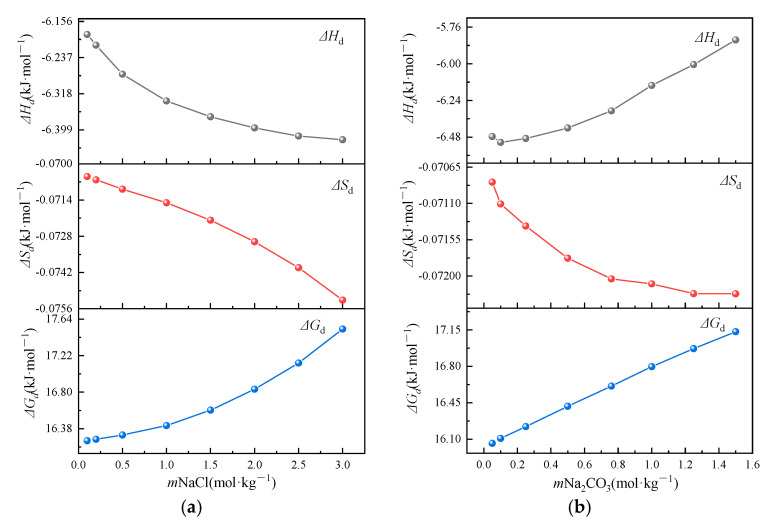
Enthalpy of dissolution (Δ*H_d_*), entropy of dissolution (Δ*S_d_*), and entropy of dissolution (Δ*G_d_*) of Li_2_CO_3_. (**a**): a mixed solution of Na_2_CO_3_ = 0.5 mol·kg^−1^; (**b**) a mixed solution of NaCl = 1.0 mol·kg^−1^.

Within the experimental temperature range, Δ*H_d_* and Δ*S_d_* are assumed to be temperature-independent based on the thermodynamic convention for limited temperature intervals. Using Equations (8) and (9), we determined the apparent molar dissolution enthalpy (Δ*H_d_*) and entropy (Δ*S_d_*) for Li_2_CO_3_ across various mixed solutions. Figure 5 presents these apparent thermodynamic parameters (Δ*H_d_*, Δ*S_d_*, Δ*G_d_*) characterizing the Li_2_CO_3_ dissolution in mixed Na_2_CO_3_-NaCl aqueous solutions, where the corresponding Gibbs free energy change (Δ*G_d_*) was calculated at the mean experimental temperature. The negative Δ*H_d_* values (Δ*H_d_* < 0) confirm the exothermic nature of the dissolution, which is consistent with the observed inverse solubility–temperature dependence. NaCl and Na_2_CO_3_ exhibit antagonistic modulations on the dissolution enthalpy: (a) at the fixed Na_2_CO_3_ (0.5 mol·kg^−1^), Δ*H_d_* decreases (becomes more negative) with the increasing NaCl concentration; (b) conversely, at the fixed NaCl (1.0 mol·kg^−1^), Δ*H_d_* increases (becomes less negative) progressively with the Na_2_CO_3_ concentration. This antagonistic effect suggests that NaCl enhances the temperature sensitivity of the solubility, whereas Na_2_CO_3_ mitigates thermal effects. The calculated Δ*H_d_* for the dissolution in the NaCl-Na_2_CO_3_ system ranges from approximately −5.8 to −6.4 kJ·mol^−1^. This value is significantly more negative than the standard dissolution enthalpy of −4.3 kJ·mol^−1^ reported in the literature [21]. The observed difference arises primarily because the dissolution medium (NaCl-Na_2_CO_3_ brine) constitutes a non-ideal solution, where significant deviations from ideal behavior occur due to ionic interactions and altered activity coefficients.

The red dashed trendline in Figure 5 delineates the molality-dependent dissolution entropy (Δ*S_d_*) evolution. Crystalline dissolution comprises two sequential processes: (i) lattice disintegration into constituent ions and (ii) subsequent ion hydration. The first stage generates increased ionic disorder, yielding positive sublimation entropy (Δ*_sub_S^θ^_m_* > 0). The subsequent hydration stage induces hydration structuring around ions, producing negative hydration entropy (Δ*_hyd_S^θ^_m_* < 0). The net dissolution entropy (Δ*_s_S^θ^_m_*) is therefore the following summation: Δ_s_*S_m_*^θ^ = Δ_sub_*S*^θ^_m_ + Δ_h_*S*^θ^_m_ [23,24]. The observed Δ*S_d_* < 0 indicates an overall entropy reduction during dissolution, signifying the dominance of the hydration entropy over sublimation effects. The ionic hydration process requires the destruction of the original ionic hydration in the solution and the establishment of a new mixed ionic hydration structure. The dissolution entropy (Δ*S_d_*) exhibits an inverse relationship with the salt molality across both NaCl and Na_2_CO_3_ concentration gradients. The progressive salt addition depletes free water molecules available for hydration. The enhanced ion association generates ordered ionic assemblies, which amplify solution structuring effects and thereby intensify negative Δ*S*_d_ magnitudes at elevated ionic strengths.

The concentration dependence of the Gibbs free energy change (Δ*G_d_*) for Li_2_CO_3_ dissolution was quantitatively evaluated using the Gibbs–Helmholtz relationship (Equation (9)). Figure 5 reveals positive Δ*G_d_* values (Δ*G_d_* > 0) across all experimental conditions, confirming the thermodynamically unfavorable nature of the Li_2_CO_3_ dissolution. The progressive increase in Δ*G_d_* with rising salt molalities demonstrates that the concomitant addition of both salts produces synergistic solubility suppression in industrial lithium precipitation systems.

**Figure 6:**
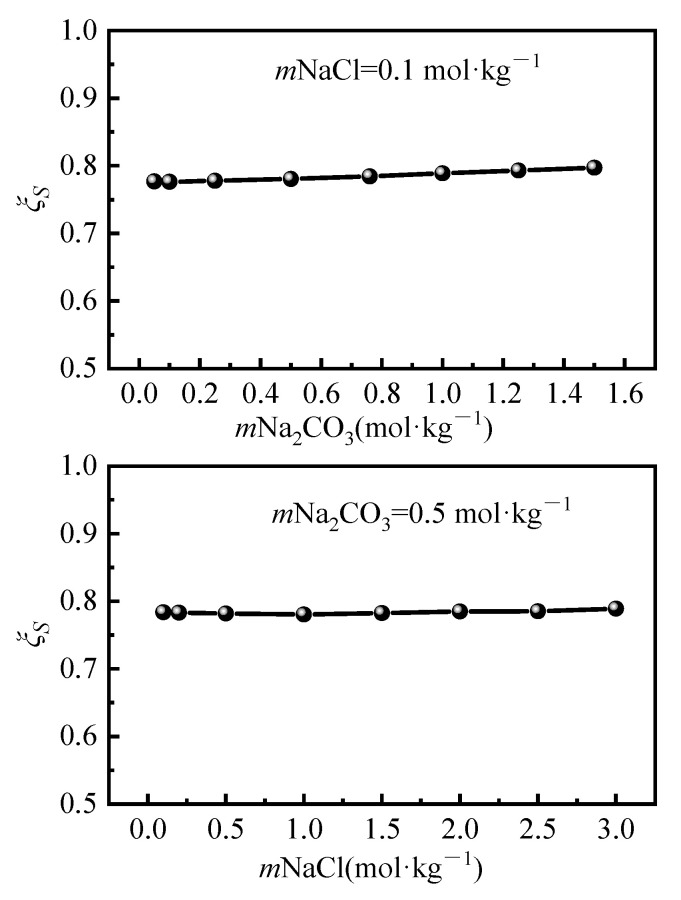
Li_2_CO_3_ dissolved entropy drive percentage versus salt molality.

The entropic contribution to the dissolution spontaneity was quantified through the dimensionless parameter: [25,26]. As shown in Figure 6, calculated *ξ_S_* values range from 0.781 to 0.797 across experimental conditions, exhibiting a positive correlation with the Na_2_CO_3_ molality. This confirms entropy-driven dominance in the Li_2_CO_3_ dissolution. The combined evidence of negative dissolution entropy (Δ*S_d_* < 0) and a high entropic contribution (*ξ_S_* ≈ 0.79) reveals that hydration structure reorganization governs dissolution thermodynamics. Specifically, Li^+^ ions exhibit exceptional hydration energy compared to other alkali cations, which intensifies water ordering effects and thereby suppresses the Li_2_CO_3_ solubility relative to its alkali metal analogs. This finding is consistent with the work of Lee et al. [21], who calculated thermodynamic dissolution parameters for M_2_CO_3_ (M = Li, Na, K) and demonstrated that dissolution entropy is the primary factor governing the solubility differences among these alkali carbonates.

## 4. Conclusions

Through isothermal dissolution equilibrium studies of Li_2_CO_3_ in lithium precipitation mother liquors across 283.15–353.15 K, with the precise determination of the equilibrium solubility, we derive the following conclusions:

(1) Concentration dependence: Li_2_CO_3_ solubility exhibits an inverse proportionality to both Na_2_CO_3_ and NaCl molalities. Notably, Na_2_CO_3_ manifests dual behaviors: while its common-ion effect dominates the solubility reduction, a concurrent weak salting-in effect partially offsets this suppression. NaCl exerts unidirectional salting-out effects through ionic strength modulation.
(2) Model validation: The Extended Debye–Hückel (E-DH) model demonstrates a strong predictive capability, yielding residual standard deviations < 0.09 across all isotherms, confirming its applicability to industrial lithium precipitation systems.
(3) Thermodynamic analysis: The systematic evaluation of dissolution parameters reveals the apparent enthalpy of the dissolution (Δ*H_d_* < 0), with positive Δ*G_d_* values confirming non-spontaneous dissolution behavior. Negative Δ*S_d_* values indicate enhanced solution ordering, with the entropy-driven percentage (*ξ_S_*) maintaining at 78% across all salt concentrations. The predominance of entropic control suggests that dissolution dynamics are governed by the hydration structure reorganization rather than enthalpic interactions.

Investigating the thermodynamic properties of lithium carbonate dissolution in lithium precipitation mother liquors can provide critical insights for optimizing industrial processes to enhance lithium recovery yields during carbonation crystallization.

## Figures and Tables

**Table 1:** Reagent description table.

Chemical Reagents	CAS No.	Initial Purity *w* (*w* %)	Purified Method	Final Purity *w* (*w* %)	Source
NaCl	7647-14-5	≥95.0%	Recrystallization	≥99.5%	Sinopharm Chemical Reagent Co., Ltd., Shanghai, China.
Na_2_CO_3_	497-19-8	≥95.0%	Recrystallization	≥99.5%	
Li_2_CO_3_	554-13-2	≥98.0%		≥98.0%	

**Table 2:** Experimental apparatus.

Instruments	Type	Source
Magnetic Stirring Thermostat	HXC-500-8A	Beijing Huicheng Jiayi Technology Co., Beijing, China
Thermostatic Water Bath	VIVO RT4	ULEB Technology (Beijing) Co., Beijing, China
Automatic Electronic Analyzing Balance	BT224S	Sartorius Scientific Instruments Co., Beijing, China
Inductively Coupled Plasma Emission Spectrometer	PerkinElmer Avio 200	PerkinElmer Inc., Wellesley, MA, USA
X-ray Diffraction instrument	D8 Discover	Bruker Co., Billerica, MA, USA

**Table 3:** Solubility of Li_2_CO_3_ in pure water (*p* = 77.3 KPa) ^a^.

*T* (K)	Solubility of Li_2_CO_3_ (Pure Water)				100 × *ε*		
	Experimental Data (mol·kg^−1^)	Literature Data [14] (mol·kg^−1^)	Literature Data [17] (mol·kg^−1^)	Literature Data [18] (mol·kg^−1^)	Literature [14]	Literature [17]	Literature [18]
283.15	0.1932	-	-		-	-	-
293.15	0.1838	0.1841	0.1801	0.18041	−0.16	2.08	−1.84
303.15	0.1689	0.1676	0.1705	0.16893	0.80	−0.93	0.02
313.15	0.1571	0.1554	0.1583	0.15648	1.11	−0.74	−0.39
323.15	0.1464	0.1487		0.14819	−1.56	-	1.22
333.15	0.1374	0.1388	0.1367	0.13706	−0.99	0.51	−0.25
343.15	0.1291	0.1310		0.12596	−1.48		−2.43
353.15	0.1168	0.1179	0.1151	0.11542	−0.97	1.51	−1.18

^a^ The standard uncertainties *u* are *u*(*T*) = 0.03 K, *u*(*p*) = 5 kPa, and *u*(*m*) for Li_2_CO_3_ is 0.0060.

**Table 4:** Solubility of Li_2_CO_3_ in mixed solutions of Na_2_CO_3_ and NaCl at 283.15–353.15 K and pressure of *p* = 77.3 kPa ^a^.

Na_2_CO_3_ (mol·kg^−1^)	NaCl (mol·kg^−1^)	Li_2_CO_3_ (mol·kg^−1^)							
		283.15 K	293.15 K	303.15 K	313.15 K	323.15 K	333.15 K	343.15 K	353.15 K
0.500	0	-	-	0.1295	0.1215	0.1141	0.1077	0.0976	0.0835
0.500	0.100	-	0.1379	0.1283	0.1204	0.1135	0.1066	0.0955	0.0827
0.500	0.200	-	0.1360	0.1270	0.1189	0.1122	0.1055	0.0943	0.0818
0.500	0.500	0.1449	0.1349	0.1227	0.1160	0.1081	0.1020	0.0916	0.0790
0.500	1.000	0.1439	0.1308	0.1144	0.1085	0.1027	0.0954	0.0861	0.0737
0.500	1.500	0.1428	0.1239	0.1045	0.0987	0.0929	0.0878	0.0794	0.0675
0.500	2.000	0.1394	0.1126	0.0932	0.0870	0.0828	0.0792	0.0707	0.0606
0.500	2.500	0.1319	0.1006	0.0803	0.0757	0.0725	0.0696	0.0611	0.0529
0.500	3.000	0.1217	0.0880	0.0660	0.0634	0.0618	0.0590	0.0520	0.0444
0	1.000	0.1091	0.0743	0.1418	0.1352	0.1229	0.1147	0.1029	0.0902
0.050	1.000	0.0966	0.1504	0.1388	0.1342	0.1207	0.1125	0.1007	0.0884
0.100	1.000	0.0823	0.1471	0.1359	0.1276	0.1186	0.1104	0.0987	0.0866
0.250	1.000	0.1584	0.1449	0.1273	0.1205	0.1124	0.1044	0.0942	0.0815
0.500	1.000	0.1550	0.1369	0.1144	0.1085	0.1027	0.0954	0.0861	0.0737
0.760	1.000	0.1528	0.1239	0.1025	0.0982	0.0933	0.0874	0.0787	0.0665
1.000	1.000	0.1449	0.1117	0.0930	0.0891	0.0854	0.0812	0.0712	0.0608
1.250	1.000	0.1319	0.1001	0.0846	0.0810	0.0778	0.0743	0.0654	0.0558
1.500	1.000	0.1196	0.0914	0.0777	0.0733	0.0710	0.0685	0.0602	0.0517

^a^ The standard uncertainties *u* are *u*(*T*) = 0.03 K and *u*(*p*) = 5 kPa. *u_r_*(*m*) for Na_2_CO_3_ and for NaCl are 0.0050 and 0.0050, respectively. *u*(*m*) for Li_2_CO_3_ is 0.0060.

**Table 5:** Parameters and standard deviations of Debye–Hückel extension model at different temperatures.

*T*/K	*A*	Na_2_CO_3_ = 0.5 mol·kg^−1^			NaCl = 1.0 mol·kg^−1^			δ
		*Bα*	*C*	*D*	*Bα*	*C*	*D*	
283.15	0.5499	36.0149	−0.1621	0.0525	7.0640	−0.4988	0.0545	0.0034
293.15	0.5212	41.9910	−0.1499	0.0534	6.9391	−0.4939	0.0544	0.0036
303.15	0.4988	34.0053	−0.1693	0.0590	7.0089	−0.4810	0.0527	0.0033
313.15	0.4759	41.4239	−0.1504	0.0555	6.4120	−0.5026	0.0555	0.0028
323.15	0.4539	85.9871	−0.1102	0.0476	5.4438	−0.5663	0.0620	0.0033
333.15	0.4336	113.7928	−0.0951	0.0442	5.1686	−0.5762	0.0620	0.0032
343.15	0.4147	50.5828	−0.1166	0.0488	4.7531	−0.6137	0.0675	0.0033
353.15	0.3971	101.3577	−0.0956	0.0459	4.8608	−0.6117	0.0674	0.0031

## Data Availability

The original contributions presented in this study are included in the article. Further inquiries can be directed to the corresponding author.
